# Dexmedetomidine Based Sedation for Post-surgery Critically Ill Adults: A Meta-analysis of Randomized Controlled Trials

**Published:** 2017-12

**Authors:** Heng FAN, Yu ZHAO, Min SUN, Ji-Hui YE, Guo-Dong CHEN, Jian-Hua ZHU

**Affiliations:** 1. Dept. of Intensive Care Unit, Ningbo First Hospital, Ningbo, China; 2. Dept. of Nephrology, Ningbo Urology and Nephrology Hospital, Ningbo, China

**Keywords:** Dexmedetomidine, Sedation, Mechanical ventilation, Delirium

## Abstract

**Background:**

Using dexmedetomidine (Dex) as a sedative agent may benefit the clinical outcomes of post-surgery patients. We reviewed randomized controlled trials (RCTs) to assess whether use of a Dex could improve the outcomes in post-surgery critically ill adults.

**Methods:**

We searched Medline, Embase, PubMed, and the Cochrane databases for RCTs comparing Dex with propofol or a placebo in post-operative patients, all included RCTs should be published in English before Jul 2016. Citations meeting inclusion criteria were full screened, and trial available data were abstracted independently and the Cochrane risk of bias tool was used for quality assessment.

**Results:**

Sixteen RCTs involving 2568 patients were subject to this meta-analysis. The use of a Dex sedative regimen was associated with a reduce delirium prevalence [odd ratio (OR):0.33, 95% confidence intervals (CI): 0.24–0.45, *I*^2^= 5%, *P*<0.001], a shorter the length of ICU stay [mean difference (MD): −0.60, 95%CI: −0.69 to −0.50, *I*^2^=40%, *P*<0.001] and the length of hospital stay [MD: −0.68, 95%CI: −1.21 to −0.16, *I*^2^=0%, *P*=0.01]. However, using of Dex could not shorter the duration of mechanical ventilation [MD: −10.18. 95%CI: −31.08–10.72, *I*^2^=99%, *P*=0.34], but could shorter the time to extubation in post-surgery patients [MD: −47.46, 95%CI: −84.63–10.67, *I*^2^=98%, *P*=0.01].

**Conclusion:**

The use of a Dex sedative regimen was associated with a reduce delirium prevalence, a shorter the length of ICU and hospital stay, and a shorter time to extubation in post-surgery critical ill patients.

## Introduction

Approximately 30% of patients suffer delirium, anxiety, and stress after surgery (1). To maintain safety and improve comfort, an optimal sedation regimen is essential for treatment of post-surgery patients in intensive care unit (ICU). A most appropriate sedative drug which long-term used in ICUs should be quick in onset and offset, cheaper, without additional adverse effects, and can be able to facilitate daily ICU procedure, reduce anxiety, improve tolerance of mechanical ventilation, shorten the length of ICU and/or hospital stay, and reduce the morbidity and mortality (2, 3). So far, there is no sedative medicine possess full of these ideal properties.

Nowadays, propofol is a preferred sedative widely used in anesthesia and ICU, which offers many advantages over benzodiazepines, such as rapid onset, easy adjustment, lack of accumulation, and quick recovery (4). Propofol has both effects of sedative and hypnotic, which mediate GABA receptor but no analgesic effect (5). Adverse reaction from propofol included respiratory depression, hypotension, hypertriglyceridemia, unpredictable duration of action, and propofol infusion syndrome (6). Moreover, propofol and benzodiazepines have also been found that might be related to the high risk of delirium (7).

Dexmedetomidine (Dex) is a relatively new agent increasingly used in anesthesia and ICU in the past decade. Dex blunts the central nervous system excitation by stimulating α-2-adrenergic receptor in the locus coeruleus (8). Compare with other sedative drugs, Dex also has other potential analgesic effects, which can reduce the incidence of delirium, shorten mechanical ventilation duration, lower hospital cost, and induce a sedation and analgesia condition close to physiologic sleep but no respiratory depression.

Although Dex has so many ideal properties for sedation in ICUs, its benefits and risks impact on outcomes of post-surgery critically ill patient, remain uncertain. In particular, some new large randomized controlled trials (RCTs) have not yet to be included in any meta-analysis. Thus, this updated meta-analysis will compare Dex with propofol or placebo in terms of the delirium prevalence, duration of mechanical ventilation, time to extubation, the length of ICU stay and adverse reaction in post-surgery critically ill adults.

## Methods

### Trial Identification

Two researchers independently conducted a literature search of Medline, Embase, PubMed, and the Cochrane databases; all included papers should be randomized controlled trials (RCTs) and published in English before Jul 2016. We only searched the studies provide the results from adults (age >18 yr old). Case reports, review, the letters, and comments were excluded from the primary search. Search keywords were “dexmedetomidine (Dex)” with “sedation”, ‘sedative agent”, “analgesia”, “critically ill”, post-operative (including post-operative, post-operation, and surgery). Only RCTs comparing Dex with propofol or a placebo were included. The trials that used Dex as anesthesia in the process of operation but continue to apply in the ICU for sedation less than 6h were excluded. Additional studies were identified according to “Google Scholar” by screening the reference lists of the related papers.

### Data Abstraction

Two investigators browsed all included studies to determine whether they fulfilled all criteria of inclusion and recorded the features and outcomes of trial by a data abstraction form independently. The primary outcome of this study was the delirium prevalence, with secondary outcomes including time to extubation, duration of mechanical ventilation, the length of ICU stay and adverse reaction. All publication RCTs were retrieved and extracted the data. Any disagreement with opinions was resolved by means of consensus with all investigators.

### Risk of Bias Assessment

Two reviewers independently conducted methodological quality assessment. The Cochrane risk of bias tool was used to evaluate the quality of included trials (9). The following seven different domains constituted the methods adequacy of sequence generation; allocation concealment; blinding of outcome assessment; blinding of participants and caregivers; incomplete outcome data and other bias. A judgment of high, low or unclear risk of material bias was made for each item according to the methods.

#### Statistical Analysis

Continuous outcomes (such as duration of mechanical ventilation and time to extubation) were calculated as mean difference (MD) with 95% confidence intervals (CI) using a random-effects model. Categorical outcomes (such as the delirium prevalence, hypotension, tachycardia, and bradycardia) were calculated as odds ratio (OR) with 95% CIs using a fixed-effects model. The heterogeneity among RCTs was evaluated using the Chi-square statistics, and the inconsistency degree was evaluated by the *I*^2^ statistic. Significant heterogeneity existed among the RCTs when *I*^2^>50%. Publication bias was evaluated by the funnel plot. The data were analyzed using Review Manager (ver. 5.2, the Cochrane Collaboration, UK, 2003), and a *P*-value<0.05 was considered as significant difference in this meta-analysis.

## Results

### Trial Identification

**Fig. 1:**
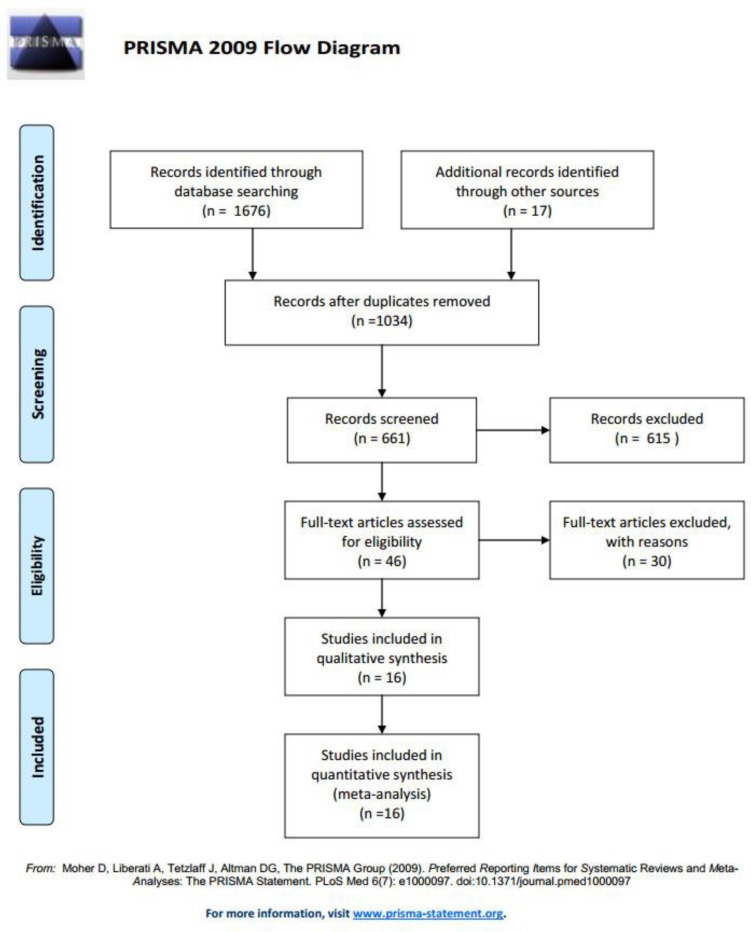
Flowchart to select the final 16 manuscripts

**Fig. 2:**
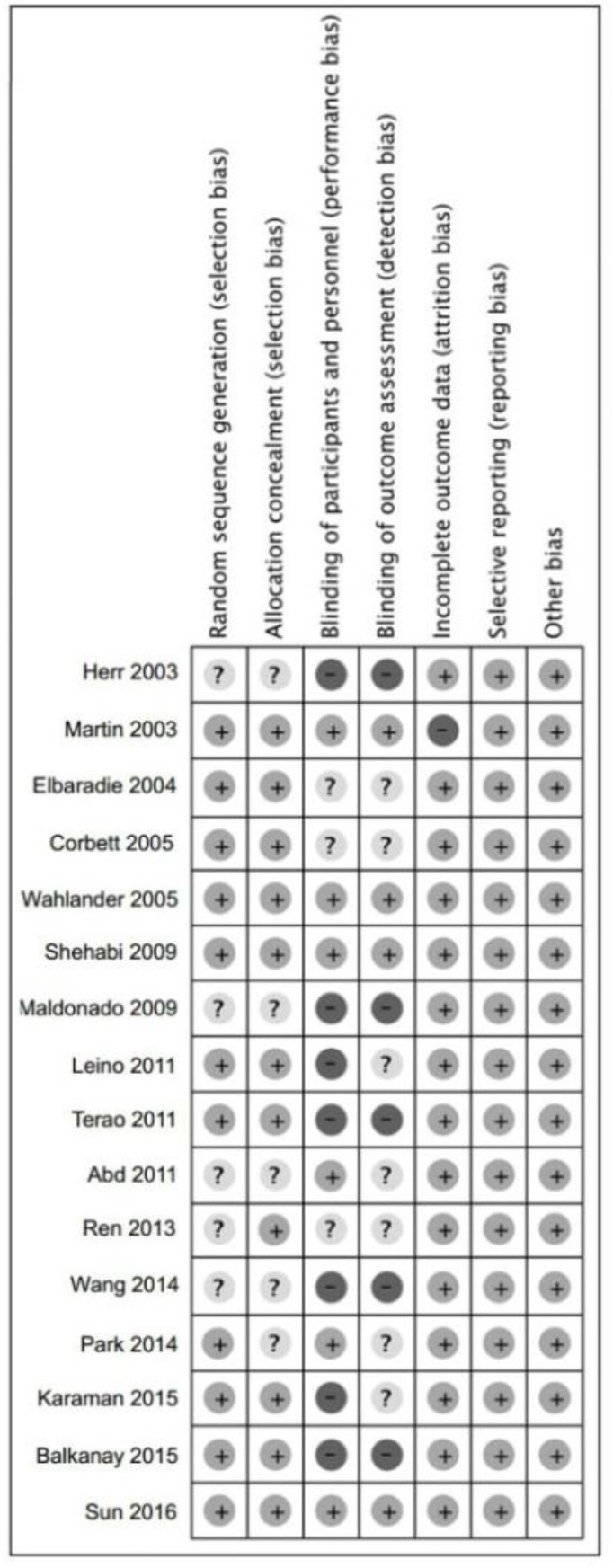
Methodological quality of trials using the Cochrane Risk of Bias Methods (+)= low risk of bias, (?)=unclear, (−)=high risk of bias

The search strategy results shown in the Fig. 1. Overall, 1637 manuscripts did not meet the criteria of inclusion or duplicates were retrieved from the four databases. We excluded the trials on bases of patient age, article type and the quality of the patients. Finally, 46 studies were fully reviewed, of which sixteen trials met all the criteria. These sixteen manuscripts involving 2568 post-surgery patients from more than ten countries were confirmed and conducted meta-analysis, and all included trials were RCTs and published in English (10–25). The Cochrane risk of bias assessment for each article is present in Fig. 2. Nine of sixteen (56.3%) trials (12–15, 25) have overall low risk of bias assessment, four trials (25%) (18–20, 24) have overall unclear risk of bias assessment, and three trials (18.7%) (10, 16, 21) have overall high risk of bias assessment.

### Trial Characteristics

The features of all included RCTs presented in Table 1, including patient number and population, Dex loading dose, Dex sustain infusion dose, experimental and control interventions, sedation levels, and outcomes used in the meta-analysis. Eleven studies enrolled post-surgery patients from two or more center, and five studies from single center (14, 18, 19, 21, 23). The age of included patients was older on average (64±23 yr old) and critically ill (average APACHE II score=23).

**Table 1:** Description of the 16 RCTs included in the Meta-analysis. Dex: Dexmedetomidine; NA: Not Applicable; RASS: Richman agitation-sedation scale; RSS: The Ramsay Sedation Scale; VAS: Visual analogue scale

***Study***	***Population***	***Dex patients***	***Control patients***	***Dex Loading Dose***	***Dex Infusion Dose***	***Comparator***	***Comparator Dose***	***Outcomes used in the meta-analysis***	***Sedation level***
Herr DL et al. 2003 (10)	Patients (y>18) after CABG surgery	148	147	1.0 μg/kg for 20 min	0.2 to 0.7 μg/kg/h	Propofol	NA	Delirium, hypotension, bradycardia, tachycardia,	RSS: 4.5
Martin E et al. 2003 (11)	Patients (y>18) requiring sedation and ventilation after surgery	203	198	1.0μg/kg for 10 min	0.2 to 0.7μg/kg/h	Placebo	1.0μg/kg	Delirium, bradycardia, tachycardia, duration of intubation, time to extubation,	RSS: 3.0–6.0
Elbaradie S et al. 2004 (12)	Patients (y>18) requiring sedation and ventilation after surgery	30	30	2.5 μg/kg/h over 10 min	0.2–0.5 μg/kg/h	Propofol	0.5–1 mg/kg/h	Time to extubation	RSS: 3.1–5.1
Corbett SM et al. 2005 (13)	Patients (y>18) requiring sedation and ventilation after CABG surgery	43	46	1.0 μg/kg over 15 min	0.4 μg/kg/h	Propofol	0.2–0.7 mg/kg/h	The length of ICU stay	RSS: 3.0–4.2
Wahlander S et al. 2005 (14)	Patients (y>18) after thoracic surgery	14	14	0.5 μg/kg over 20 min	0.4 μg/kg/h	Placebo	0.4 μg/kg/h	Hypotension	VAS: 0.7–3.9
Shehabi Y et al. 2009 (15)	Patients (y>60) after cardiac surgery	152	147	NA	0.49 μg/kg/h	Placebo	49 μg/kg/h	Delirium, bradycardia, tachycardia, the length of ICU stay	NA
Maldonado JR et al. 2009 (16)	Patients (y>18) after cardiac surgery	40	38	0.4 μg/kg once intravenous injection	0.2–0.7 μg/kg/h	Propofol	25–50 μg/kg/min	Delirium, the length of hospital stay, the length of ICU stay	NA
Leino K et al. 2011 (17)	Patients (y>21) after CABG surgery	44	43	1.0 μg/kg for 20 min	0.2–0.5 μg/kg/h	Placebo	0.2–0.5 μg/kg/h	Time to extubation	NA
Terao Y et al. 2011 (18)	Patients (y>18) requiring sedation and ventilation after surgery	16	16	0.1 μg/kg for 10 min	0.4 μg/kg/h	Propofol	1.0 mg/kg/h	Duration of intubation, the length of ICU stay	RSS: 2.0–6.0
Abd N et al. 2011 (19)	Patients (y>18) requiring sedation and ventilation after surgery	14	14	4 μg/kg once intravenous injection	0.03–0.25μg/kg/h	Placebo	0.4–0.6μg/kg/h	Time to extubation	RSS: 2.0–3.0
Ren JJ et al. 2013 (20)	Patients (y>18) after CABG surgery	81	81	NA	0.2–0.5 μg/kg/h	Propofol	2–4 mg/kg/h	Tachycardia	NA
Wang ZX et al. 2014 (21)	Patients (y>18) after hepatectomy	22	22	1 μg/kg over 10 min	0.3 μg/kg/h	Propofol	3–4 mg/kg/h	Duration of intubation, the length of hospital stay	NA
Park JB et al. 2014 (22)	Patients (y>18) after CABG surgery	67	75	0.5μg/kg once intravenous injection	0.2–0.8 μg/kg/h	Placebo	0.4–0.6μg/kg/h	Delirium, time to extubation, the length of ICU stay, the length of hospital stay, bradycardia	RASS: −2.0-0
Karaman Y et al. 2015 (23)	Patients (y>18) after CABG surgery	31	33	NA	0.6 μg/kg/h	propofol	2 mg/kg/h	Time to extubation, hypotension, bradycardia, tachycardia	RSS: 2.0–3.0
Balkanay OO et al. 2015 (24)	Patients (y>18) after CABG surgery	31	28	4 μg/kg once intravenous injection	0.04μg/kg/h	Placebo	0.04μg/kg/h	Delirium, hypotension, bradycardia, duration of intubation, the length of ICU stay, the length of hospital stay	RSS: 2.0–3.0
Sun X et al. 2016 (25)	Patients (y>65) after non-cardiac surgery	350	350	NA	0.1μg/kg/h	Placebo	0.1μg/kg/h	Delirium, hypotension, bradycardia, tachycardia, time to extubation, the length of ICU stay, the length of hospital stay	RASS: −2.0-0

The largest study contained 700 post-surgery patients (25), whereas the smallest study included 28 post-surgery patients (14, 19). Ten trials studied critically ill patients after cardiac and vascular surgery (10, 13, 15–17, 19, 20, 22–24), and five trials studied non-cardiac surgery (12, 14, 18, 21, 25), and one trial studied the patient after cardiac and non-cardiac surgery. Eight trials compared Dex with placebo, and eight studies compared Dex with propofol. A loading dose of Dex was used in twelve studies. The maximum maintenance doses of Dex ranged 0.2 to 0.7μg/kg/h. With except one study routinely monitored sedation (14), all trials included an established goal.

### Clinical outcomes

**Fig. 3:**
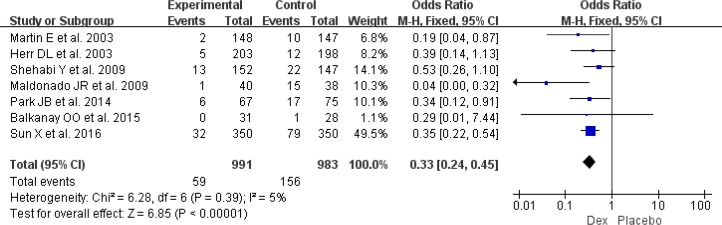
Meta-analysis of postoperative delirium prevalence. *df* = degrees of freedom, *M-H* = Mantel-Haenszel

Seven RCTs reported delirium prevalence as an outcome (n=1894), the use of a Dex sedative regimen was associated with a reduce delirium prevalence (10, 11, 15, 16, 22, 24, 25) (OR:0.33, 95% CI: 0.24–0.45, *I*^2^= 5%, *P*<0.001) (Fig. 3).

**Fig. 4:**
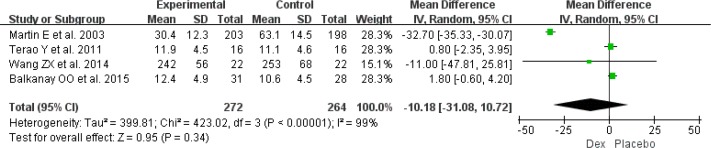
Meta-analysis of duration of mechanical ventilation. *df* = degrees of freedom

**Fig. 5:**
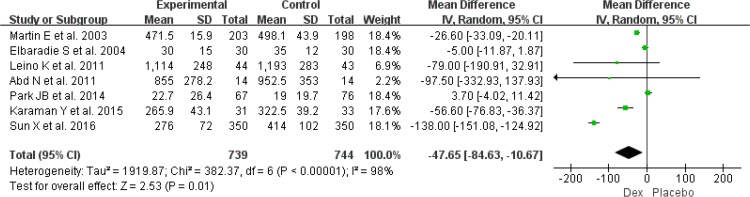
Meta-analysis of time to extubation. *df* = degrees of freedom

Significant heterogeneity existed in duration of mechanical ventilation (*I*^2^=99%) and time to extubation (*I*^2^=98%) among the included RCTs, and duration of mechanical ventilation was found from four RCTs involving 536 patients (11, 18, 21, 24). When pooled, using of Dex could not shorten the duration of mechanical ventilation (MD: −10.18, 95%CI: −31.08–10.72, *I*^2^=99%, *P*=0.34) (Fig. 4), but the use of Dex was associated with a shorter of time to extubation in post-surgery patients (MD: −47.46, 95%CI: −84.63–10.67, *I*^2^=98%, *P*=0.01) (Fig. 5).

**Fig. 6:**
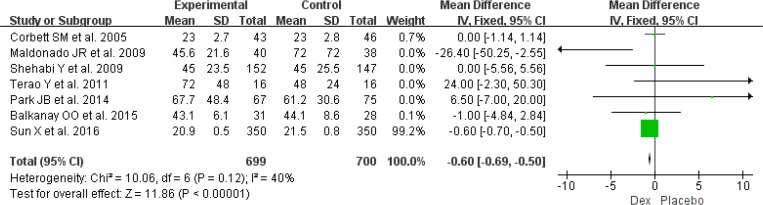
Meta-analysis of the length of ICU stay. *df* = degrees of freedom

Data from seven RCTs (n=1399) found that use of Dex was associated with a shorter the length of ICU stay (13, 15, 16, 18, 22, 24, 25) (MD: −0.60, 95%CI: −0.69 to −0.50, *I*^2^=40%, *P*<0.001) (Fig. 6).

**Fig. 7:**
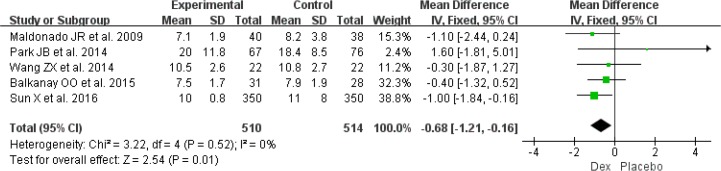
Meta-analysis of the length of hospital stay. *df* = degrees of freedom

Result from five RCTs indicated that use of Dex was also associated with a shorter the length of hospital stay (16, 21, 22, 24, 25) (MD: −0.68, 95%CI: −1.21 to −0.16, *I*^2^=0%, *P*=0.01) (Fig. 7).

**Fig. 8:**
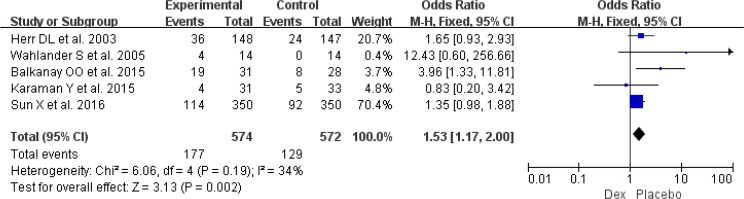
Meta-analysis of postoperative hypotension. *df*=degrees of freedom, *M-H*=Mantel-Haenszel

**Fig. 9:**
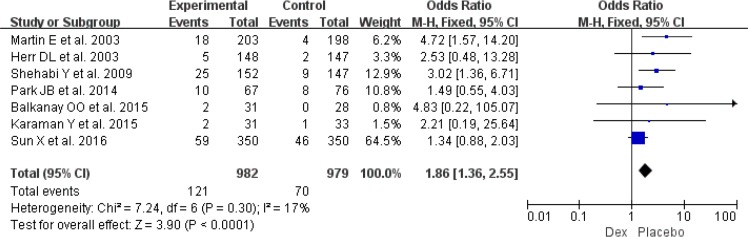
Meta-analysis of postoperative bradycardia. *df*=degrees of freedom, *M-H*=Mantel-Haenszel

**Fig. 10:**
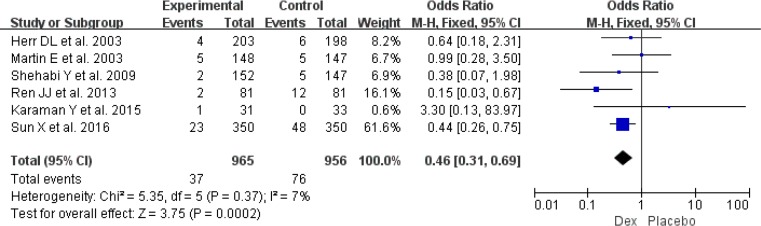
Meta-analysis of postoperative tachycardia. *df* = degrees of freedom, *M-H* = Mantel-Haenszel.

Postoperative hypotension was available from five RCTs involving 1146 patients (10, 14, 23–25). Risk for hypotension (OR: 1.53, 95% CI: 1.17–2.00, *I*^2^=34%, *P*=0.002) (Fig. 8) was significantly higher between Dex and propofol or placebo regimens requiring interventions. Moreover, seven RCTs involving 1961 patients provided the data of bradycardia (10, 11, 15, 22–25), and use of Dex could increase the risk of bradycardia (OR: 1.86, 95% CI: 1.36–2.55, *I*^2^= 17%, *P*<0.001) (Fig. 9). However, result from six RCTs showed that use of Dex was associated with reduction of tachycardia (10, 11, 15, 20, 23, 25) (OR: 0.46, 95% CI: 0.31–0.69, *I*^2^= 7%, *P*<0.001) (Fig. 10).

**Fig. 11:**
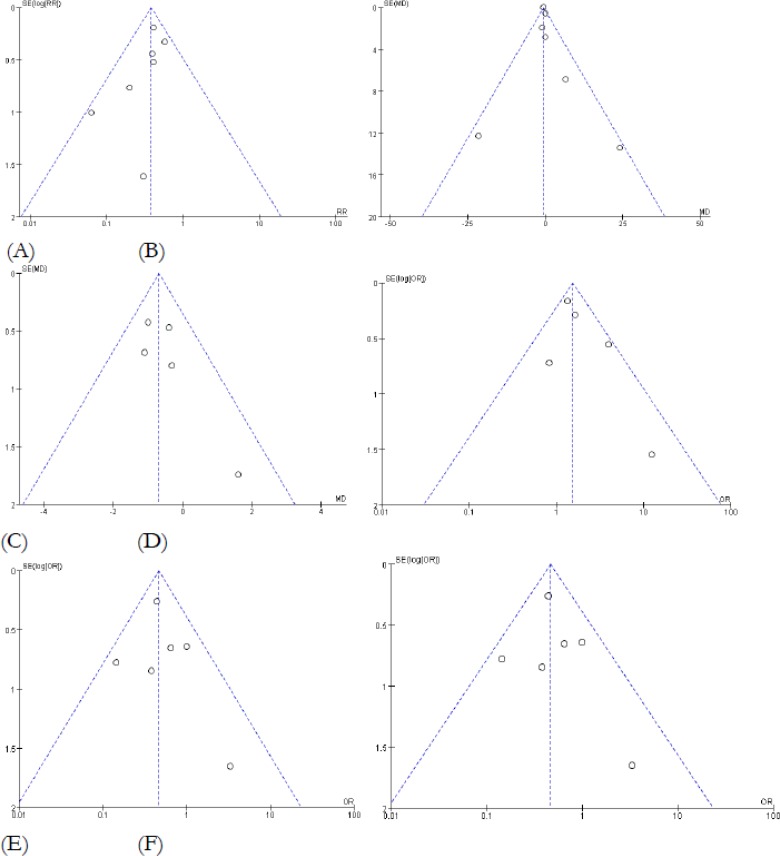
Funnel plot of meta-analysis of Dex based sedation for postoperative patients. (A) Postoperative delirium (Begg’s test, *P*=0.172; Egger’s test, *P*=0.208); (B) The length of ICU stay (Begg’s test, *P*=1.000; Egger’s test, *P*=0.900); (C) The length of hospital stay (Begg’s test, *P*=0.806; Egger’s test, *P*=0.900); (D) Hypotension (Begg’s test, *P*=0.221; Egger’s test, *P*=0.179); (E) Bradycardia (Begg’s test, *P*=0.548; Egger’s test, *P*=1.92); (F) Tachycardia (Begg’s test, P=0.707; Egger’s test, *P*=0.275). SE: standard error; MD: mean difference; OR=odds ratio.

Funnel plot, as well as Begg’s and Egger’s tests, were conducted to assess publication bias of trials. There was no publication bias in postoperative delirium, the length of ICU stay, the length of hospital stay, hypotension, bradycardia and tachycardia (Fig. 11).

## Discussion

In this meta-analysis, sixteen RCTs met the inclusion criteria and used to assess the effects of Dex on the outcomes of post-surgery critical ill adults. The results suggested that the use of Dex in post-surgery patients was associated with reducing delirium prevalence, 0.60 d shorter length of ICU stays, 0.68 d shorter length of hospital stay, 47.46 h shorter time to extubation. The use of Dex was associated with increased risk of hypotension and bradycardia, but reduced risk of tachycardia.

The potential favorable pharmacologic characteristics of Dex could help to decrease the risk of delirium in critically ill patients, but the results of these studies remained controversial (26, 27). In our study, we involved seven RCTs and found a decreased risk of delirium after using Dex for post-surgery critically ill patients. Dex sedation was associated with reduced morbidity of delirium in critically ill patients with post-cardiac surgery (28, 29). Contrarily, using Dex in ICU patients had not decrease the risk of delirium in their results of meta-analysis (30).

The different results of meta-analyses for risk of delirium might be included following reasons. Firstly, some included RCTs needed to adjust sedative agents to reach the targeted sedation level. However, many studies reported different risk of delirium and by different assessment scales of sedation. Secondly, most included studies titrated the dose of Dex on the basis of required sedation level, but no one study used mandatory daily sedation interruption to avoid over-sedation. Thirdly, some included RCTs excluded critically ill patients with neurological diseases and cannot communicate.

Dex was very effective in decrease the length of ICU stay, therefore, Dex might be more functional for critically ill patients as a supplementary therapy (31). Our results also indicated that use of a Dex was able to reduce significantly the length of ICU stay in post-surgery critical ill patients. Dex did provide more advantages than traditional sedative agents. As well as results of published meta-analyses previously confirmed the using of Dex could reduce the length of ICU stay (32), but for post-surgery critically ill patients this was the first meta-analysis to our knowledge.

The increased risk of bradycardia with Dex was well agreed with the results of a meta-analysis based on post-cardiac critically ill patients (33). However, our meta-analysis also showed that use of Dex could decrease risk of tachycardia (Fig. 10). The increased risk of bradycardia was lead to increased risk of hypotension (Fig. 8), as thus need interventions, such as decreasing the Dex infusion rate, fluid resuscitation, using vasopressors (34). New and large RCTs included might be able to explain the reason of different findings between two meta-analyses.

This meta-analysis has several limitations. Firstly, our included RCTs involved post-cardiac surgery and post non-cardiac critically ill patients, and some included RCTs were small sample and single center. Therefore, these factors might lead to a relative overestimation of results in small trials. Secondly, for Dex interventions, variation greatly of the dose and duration among studies might generate different effects. Thirdly, the study shows that sedation protocols significantly affects long-term prognosis of critically ill patients (35). Fourthly, our meta-analysis just included the English language paper, so publication bias may exist. However, no study was designed to show a statistical difference in ICU and/or hospital mortality, and there was no pooled RCT reported about long-term prognosis about critically ill patients, so Dex effected long-term prognosis remain uncertain.

## Conclusion

The use of a Dex sedative regimen was associated with a reduce delirium prevalence, a shorter the length of ICU and hospital stay, and the use of Dex was association with a shorter of time to extubation in post-surgery critical ill patients. Moreover, Dex treatment might increase the risk of hypotension and bradycardia but decreased the risk of tachycardia. More large RCTs are needed to further clarify which kinds of post-surgery critically ill patients can gain maximum benefit from using Dex as a primary sedative agent.

## Ethical considerations

Ethical issues (Including plagiarism, informed consent, misconduct, data fabrication and/or falsification, double publication and/or submission, redundancy, etc.) have been completely observed by the authors.
